# Medico-Legal

**Published:** 1907-11

**Authors:** E. S. McKee

**Affiliations:** Cincinnati


### PROGRESS IN MEDICAL SCIENCE.

### Medico-Legal

By E. S. McKEE, M.D., Cincinnati.

### CAN'T COLLECT COMMISSIONS ON PRESCRIPTIONS.

SOME of us have been shocked by physicians asking and pharmacists offering commissions on prescriptions. The most surprising thing of all, however, is the fact of physicians advertising their method of petty grafting. Two physicians recently have entered suit for a 25 per cent. rake off from druggists which it seems had been promised them. One of these suits was entered in Racine, Wisconsin, and the other in Benares. They were both disallowed however, both judges claiming that an agreement of this sort was illegal and against public policy.

### IS CRIME INFECTIOUS.

CRIME is as infectious as smallpox, says Dr. Funk, the eminent New York psychologist, and, he adds, "unless immediate radical steps are taken to put a stop to the epidemic of crime which is raging in New York, the whole country will become infected with the desire for murder and an epoch of bloodshed will follow." The doctor recommends quick trials and justice of the severest type. He attributes the cause and increase of so much crime to the yellow press and the influx of so many immigrants from the European countries noted for their hot headedness. The infectiousness of crime is due to "unwritten law," "brain storms," insanity that gives a license to would be criminals to commit a crime, hoping that they will be acquitted on the ground of their insane condition. The thing we need to subdue crime is quicker and severer punishment.

### MEDICAL RESPONSIBILITY.

A PARIS physician had cured a case of appendicitis with the application of ice but a superficial eschar resulted, presumably the result of the ice treatment. The patient though cured entered suit for 50,000 francs damages. The court held that inattention to the rules of prudence, habitual negligence and ignorance should make the physician responsible in every case, but since the slight inconvenience was the result of an approved method of treatment belonging to the province of medical science, and was not the outcome of negligence on the part of the physician it could not hold him responsible. This is a fair rendering of the question. Is a physician responsible for results when they are through no fault of his own? The court rendering this decision was Le Tribunal Correctionelle de Paris.

### EXTRA COMPENSATION FOR MEDICAL EXPERTS.

THIS question has been decided recently in Missouri, in the case of Burnett vs. Freeman, Kansas City, Missouri Court of Appeals. Plaintiff, a practising physician in Kansas City, had been called as a witness in two suits by the defendants in which they were plaintiffs on account of alleged personal injuries. The doctor brought suit claiming $50.00 in each case for his services as an expert witness. The court held that he could not obtain extra compensation for the testimony given as an expert though if required to give services for the party calling him he could require extra compensation. Further, the court held that an agreement to pay an expert extra compensation for his testimony which he was required to give under subpoena was against public policy in Missouri. Here we have for the first time in Missouri, squarely decided, a question of the utmost importance to the medical expert, his legal right to demand from a party calling him to testify as an expert, extra compensation for his services so rendered. Rodgers on expert testimony says that the cases are about evenly balanced for and against the allowance of extra fees for expert testimony. It is claimed by those who argue for the extra compensation for experts that taking their opinions and knowledge for nothing is like taking a man's property from him. There are many instances where a medical man has devoted his whole life for the benefit of his fellows but no one has claimed that he may be compelled to do so. A physician could not claim as an expert in any other calling. If this question is once decided that the medical expert may demand an extra compensation then what is to hinder the mechanic or farmer or plumber or whitewasher to do the same thing. The court grants that if the physician must perform extra services for either litigant, as the making of a post mortem, an operation or analysis, he may charge him for these extra services.

---

### DENTISTS "JAWING" DOCTORS.

AT the instigation of several dentists a doctor has been arrested in Minneapolis charged with violating the law by pulling the teeth of a patient. The dentists allege that while a doctor is licensed to perform surgical operations it does not include the right to practise dentistry, which they aver is a separate and distinct branch of medicine allotted to them alone. They declare that a doctor who attempts to fill or extract a patient's teeth not only oversteps professional ethics, but is also violating the law and they propose to make a test case of the present one in order to establish their legal rights and to establish a precedent for all time. It looks like the complaining dentists were straining at a fine point in ethics. No one imagines that the physicians generally are anxious to enter the field allotted to dentists and the fear of competition is therefore rather far fetched. In exceptional cases it might be quite a genuine accommodation to find physicians ready and willing to remove a tooth and in such cases there should be no obstruction to the suffering patient obtaining relief. If the dentists draw the line too fine the physicians may find a way to retaliate by charging the dentists with overstepping their rights in certain procedures for relieving pain and reducing swelling. It is, in other words, a rule which might work both ways. Strange that it is not considered proper for a medical man to draw a tooth but if a part of the jaw is to come away with it, it would be perfectly proper for him to do it. What is the good of all this "jawing" anyhow.

---

### LICENSED PHARMACIST, NECESSARILY PRESENT AT SALE OF MEDICINE.

TINCTURE arnica, tincture iodine and spirits of camphor were compounded by a licensed pharmacist and placed on a counter in bottles sealed and labeled. While the only licensed pharmacist in the establishment was in the laboratory across the street a sale was made by an unlicensed clerk. Action was brought against the pharmacist for selling medicines in his store without the presence of a licensed pharmacist. The lower court gave a verdict for the druggist but the decision was reversed in the supreme court, which thought that one registered pharmacist should be in the store every minute during business hours. The court thought that the health of the community and the public welfare could not be threatened in any more perilous way than by the sale of medicines by inexperienced persons.

---

### SUBSTITUTION IN PRESCRIPTIONS AS A PENAL OFFENSE.

IN France, Germany and Denmark and probably in other European countries the offense of tampering with physicians' prescriptions constitutes a crime visited by severe punishment. In many instances the guilty parties are prosecuted and brought to justice by their fellow pharmacists, or a body to whom this duty is delegated by the pharmaceutical associations. In New York City the criminal law has been invoked to punish tampering with physicians' prescriptions. Most honest pharmacists and physicians are decidedly of the opinion that tampering or wilful substitution in a physician's prescription of any ingredient or ingredients other than those specified, for the purpose of gain and without the knowledge of either the physician or patient, is distinctively a criminal act, that it should be punished as such and that such states which have not already made such provisions on their statute books should do so at the earliest possible opportunity.

A prescription is an order from a physician to any pharmacist to compound certain drugs named therein, in proportions specified, and to deliver the same when compounded to the bearer. The pharmacist must follow his instructions explicitly. He is without discretion in the matter. He does not see the patient, does not know his needs nor idiosyncracies nor the peculiarities of his surroundings or occupation. The slightest change may upset the calculations of the physician, defeat his skill and put the patient's life in jeopardy. Let no honest druggist beguile himself into the belief that substitution does not exist or if at all present it is in such a small extent as to be of no importance. Every physician and druggist can scarcely be blind to the fact that it is alarmingly present; prevalent investigation is all that is necessary to prove it. The druggist who substitutes robs the manufacturer of the article ordered but not supplied, but that is not the worst, he abuses the confidence of the physician whose skill and reputation depend on the strict compliance with his instructions. He trifles with the health and life of the patient and strikes, gives a stab as insidious as it is deadly, at every honest pharmacist in the land.

Lawyers try to drive out the shysters from their ranks. Physicians are constantly trying to expel the quacks, merchants try to expose dishonest competitors. Honest pharmacists, who are made to suffer not only in their reputations but from unfair advantages suffer not only in their reputations but from unfair advantages in trade, which the substitutor gains by his substitution of cheaper articles, must or should in self-defense alone catch the substitutor wherever they can and brand him so that his infamy may be known to all men.

A prescription is an order from a physician to a druggist to compound certain drugs named therein, in the proportions specified and deliver the same to the bearer. The pharmacist is not unlike the paying teller in a bank. A check is presented at his window for so many dollars and cents in United States of America money. He must deliver that amount, no more, no less. He must not pay it in part counterfit money nor in part English money which is "just as good." If the check is incorrectly drawn he must refuse to pay it till the mistake is corrected.

---

### THE PHARMACIST AND VENEREAL DISEASES.

Although syphilis has long been considered a serious disease, gonorrhea has but recently been considered as serious, especially in its results. Formerly any drug clerk was considered good enough to treat a case of clap or a cold and the two troubles were by many, and are still by some, considered to be on a par. It is now agreed that instead of a drug clerk prescribing over the counter for a case of clap this disease demands the best treatment from a careful expert. What is true of gonorrhea is certainly true of syphilis. The druggist is committing a crime against his customer who presumes to treat these cases himself either by his own or patent or proprietary remedies. It is his duty to tell the patient the need of proper medical advice. True, many doctors are themselves ill prepared to guide these cases to recovery but that is not the drug clerk's fault. And doctors discuss these cases in a flippant manner and fail to impress their patients with the importance of proper treatment and prolonged care and watchfulness. Every case of venereal disease should come at once under the care of a physician, and not only that, but a competent physician, better a recognised specialist and an honest one. Gonorrhea from being considered as serious as a bad cold has become recognised every where by intelligent physicians as a most serious disease to treat and difficult to cure. There are many authorities who consider it positively incurable. They say it is apparently cured only to break out again in after years and affect the patient's wife and children. We have resulting blindness, deafness, pelvic inflammation, sterility, tuberculosis, inflammations on the brain and spinal cord, and many more sad and distressing sequelæ more or less directly dependent on this insidious disease. Druggists should not put up and push remedies of their own for either gonorrhea or syphilis and should discourage, if not absolutely refuse, to sell proprietary remedies for them. The druggist could do much to educate the people as to the seriousness of venereal diseases, for a large percentage of the cases apply first to the drug clerk. In every possible way and by every possible means the ignorance, which is criminal, should be supplanted by the knowledge which is virtuous

---

### THE WIZARD OF THE WORLD IN WINDSUCHT.

SUCH is the title given Dr. Carl Spengler, of Davos Platz, Switzerland. By long and tedious work he has developed some facts of great interest pertaining to the life, history and nature of tubercle bacilli. He has undoubtedly developed a method of differentiating human from bovine tubercle bacilli. He has demonstrated that human bacilli in tuberculosis have a spore stage and develop the bacilli from these spores or aplitter, as he first named them. He is able to grow heavy cultures of tubercle bacilli, both of the human and bovine type, in from eight to ten days, such as usually require from six weeks to two or three months to develop. From these studies Dr. Spengler has developed a theory which promises to be the best line of treatment yet undertaken.

